# Intraguild predation in sympatric seals and the effect on a declining population

**DOI:** 10.1111/1365-2656.70152

**Published:** 2025-10-12

**Authors:** Izzy Langley, Andrew Brownlow, Debbie J. F. Russell

**Affiliations:** ^1^ Sea Mammal Research Unit, Scottish Oceans Institute University of St Andrews St Andrews Fife UK; ^2^ Scottish Marine Animal Stranding Scheme, School of Biodiversity, One Health and Veterinary Medicine, College of Medical, Veterinary and Life Science University of Glasgow Glasgow UK

**Keywords:** cannibalism, citizen‐science, corkscrew lacerations, intraguild predation, Leslie matrix, population decline, predator pit, strandings

## Abstract

1. Evidence of intraguild predation (IGP) is found across taxonomic groups but varies in its prevalence within predator populations. The effects of IGP can be similar to traditional predation, but when exploited by few, specialist predators the impact on intraguild prey populations is less clear.
2. Grey seals (*Halichoerus grypus*) and harbour seals (*Phoca vitulina*) are marine top predators with similar diets of a broad range of fish and cephalopods. However, in recent years, adult male grey seals have been observed predating both grey seals and harbour seals.
3. Combining 11 years of strandings data and direct observations of predation from citizen‐science across Scotland, we examined the prevalence, spatial extent and temporal trends of IGP by grey seals. These results informed realistic predation scenarios and the effect of IGP on a small, protected population of harbour seals was explored.
4. IGP was geographically widespread, involving multiple, seemingly specialist adult males across distinct subpopulations. The prevalence of IGP, as revealed by strandings data, appeared to increase through the study. Predation was most pronounced on adult harbour seals during the breeding season, amplifying the population level impact of each predation event through the loss of future reproductive potential. Indeed, in a depleted population in southeast Scotland, the estimated peak predation level was projected to cause declines of 7%–11%, well within the rate of the current harbour seal decline.
5. This study demonstrated the utility of integrating disparate datasets to address conservation challenges and highlights how IGP, while seemingly rare, can hold sympatric species in a predator pit and in small populations may contribute to declines.

## Graphical abstract

This study examined the prevalence, spatial extent, and temporal trends of grey seal intraguild predation (IGP). We demonstrated the utility of integrating disparate datasets to address conservation challenges and highlighted how IGP, while seemingly rare, can hold sympatric species in a predator‐pit and in small populations may contribute to declines.

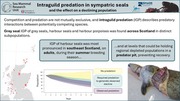

## 1 INTRODUCTION

Sympatric species have the potential to interact through interspecific competition and predation (Krebs, 1994). These interactions have top‐down and bottom‐up effects on the structure of ecological communities (Birch, 1957; Gallant et al., 2019; McPeek, 2019). Competition and predation are not mutually exclusive, and intraguild predation (IGP) describes predatory interactions between potentially competing species (Polis & McCormick, 1987). While feeding on potential competitors provides nutritional benefits to intraguild predators, it can have a secondary effect of minimizing pressures imposed through competition (Polis et al., 1989). The impact on intraguild prey populations is therefore likely to be more complex than competition or predation alone (Polis et al., 1989).

IGP is found across taxonomic groups, but the effect on intraguild prey populations varies depending on the prevalence of IGP as a specialization (Kang & Wedekin, 2013). For example, if it is a strategy exploited by individuals throughout the population, IGP can affect prey population dynamics by reducing density in populations not at density dependence (Stevens, 2010). In terrestrial carnivore communities, IGP is known to result in mesopredator suppression, but mesopredators can evade predation by spatiotemporal avoidance (Fedriani et al., 2000; Hayward & Slotow, 2009; Johnson & VanDerWal, 2009). There is also evidence to suggest that a small number of specialist predators can remove a high number of intraguild prey (Votier et al., 2004). IGP as a specialist strategy, therefore, has the potential to be significant on intraguild prey populations, especially if individuals with high reproductive value are targeted (Fisher, 1930).

IGP can occur within trophic levels, and between conspecifics, that is, intraspecific predation, or cannibalism (Fox, 1975). The drivers behind such behaviour are varied, and unlike infanticide (e.g. Packer & Pusey, 1983), the subsequent feeding on intraguild prey is less well understood but has to provide some nutritional benefit (Polis et al., 1989). When IGP is rarely observed and/or associated with a limited number of individuals, the behaviour is assumed to be aberrant and to have negligible population impact. There is also some evidence that it is a property of a system in a state of flux. For example, Steller sea lion (*Eumetopias jubatus*) predation of harbour seals (*Phoca vitulina richardii*) in Alaska has been attributed to the rapid expansion of sea lion populations which increased the frequency of adverse interspecific interactions (Mathews & Adkison, 2010). Similarly, increased walrus predation of phocid seals in the Pacific was correlated with changes in sea ice extent (Lowry & Fay, 1984). Such IGP could therefore diminish with the frequency of interspecific interactions if intraguild prey populations fall below a certain threshold and/or decrease spatial overlap with predators.

In the North Atlantic, there has been one published account of an adult male grey seal (*Halichoerus grypus*) predating young harbour seals (*P. v. vitulina*; van Neer et al., 2015). There have also been observations of grey seal cannibalism (Bédard et al., 1993; Bishop et al., 2016; Kovacs et al., 1996) and IGP of harbour porpoises (*Phocoena phocoena*; Bouveroux et al., 2014; Stringell et al., 2015). All three of these species eat a similar and broad range of fish and cephalopod species (Santos & Pierce, 2003; Wilson & Hammond, 2019). Our current understanding of predatory interactions between the species is limited to these few observations of IGP and cannibalism events, and the pathology of recovered carcasses (Brownlow et al., 2016; Haelters et al., 2012; Leopold et al., 2015; van Bleijswijk et al., 2014; Westphal et al., 2023). Drivers behind grey seal IGP are unclear, but analysis of carcasses has revealed that grey seals only consume a small section of blubber (Brownlow et al., 2016), which would suggest that IGP alone is not a sustainable alternative to the grey seal diet.

As grey seal populations throughout the North Atlantic are either increasing or approaching carrying capacity (Hammill et al., 2023; Russell et al., 2022), it is possible that grey seal IGP is an emergent behaviour due to increased competition, both intraspecifically within grey seals and interspecifically with harbour seals and harbour porpoises. Scotland hosts an increasing population of grey seals, a quarter of the global abundance (Thomas et al., 2019). Harbour seals in Scotland represent a quarter of the European holdings, and in the last 20 years, numbers on the north and east coasts have declined and have either stabilised at depleted levels or are still in decline (Thompson et al., 2019). The rate of the harbour seal decline was highest in the Firth of Tay and Eden Estuary Special Area of Conservation (SAC) in southeast Scotland, where harbour seal numbers dropped by ~95% between 2002 and 2017 (Thompson et al., 2019). It is therefore of interest to better understand the potential drivers behind these declines, which are likely complex but include adverse interspecific interactions with grey seals.

Grey and harbour seals come into direct contact both at mixed species haulout sites on land (Thompson et al., 1996) and in foraging areas at sea (Carter et al., 2022). However, in the United Kingdom they have asynchronous breeding seasons; grey seals breed in autumn/winter (Russell et al., 2019) and harbour seals breed in summer (Thompson & Wheeler, 2008). The prevalence of grey seal IGP and its effect on prey populations has never before been quantified, and it is not clear how many individuals exploit this behavioural and dietary niche. The aim of this study was to quantify the spatial and temporal extent of grey seal IGP around Scotland and investigate the potential role this could have had in harbour seal population declines. In this study, we also included individual grey seals predating grey seals and harbour porpoises because they likely represent marine mammal predating specialists, as has been found in other taxonomic groups (e.g. Lowry & Fay, 1984).

## 2 MATERIALS AND METHODS

### 2.1 Data collection

#### 2.1.1 Strandings data

In Scotland, dead marine mammals (i.e. strandings) are collected and assessed by the Scottish Marine Animal Stranding Scheme (https://strandings.org). Strandings reports are opportunistic, relying on observations from members of the public. These reports are then triaged to determine the appropriate response, which may include necropsy, sampling, or basic documentation through images and morphometrics. The extent of subsequent investigation depends on logistical constraints, the condition of the carcass and the likelihood that a necropsy will yield meaningful information to establish a cause of death. The majority of seal cases are assessed by photographs submitted via the volunteer strandings network. Experienced pathologists assess these photographs for the cause of death and assign adjectival scores described in Brownlow et al. (2016) based on the likelihood that the cause of death was grey seal predation. The adjectival scores range from *definite*, *likely*, *probable*, *possible* to *unlikely*. The *definite* score is reserved for directly observed predation cases and is therefore rare. If the pathology is most consistently explained by grey seal predation, cases are assigned as *likely* if no other causes of death are plausible, or *probable* if other causes of death are plausible. *Possible* grey seal predation cases include those where the pathology would not be inconsistent with grey seal predation. Harbour porpoise carcasses with pathological attributes indicative of grey seal predation are given a single adjectival score of *possible* grey seal predation.

#### 2.1.2 Direct observations of predation events

This study included observations of *possible* grey seal predation events by or reported to the Sea Mammal Research Unit, including those from Bishop et al. (2016). For this study, we created and publicised a reporting webpage (https://www.smru.st‐andrews.ac.uk/sealpred/) to formalise collection of these opportunistic observations, enabling contributors to submit information and media of events. Guidelines issued by the University of St Andrews School of Biology Ethics Committee for the collection, storage and analysis of citizen science data were followed and ethical approval was granted (reference BL15073). Observational reports were classified as *definite* grey seal predation if the entire interaction was captured in photographs and/or videos (both kill and subsequent feeding), and *likely* if the kill was not captured and so carcass scavenging could not be ruled out to explain the feeding behaviour; a conservative approach as there is no confirmed evidence of marine mammal scavenging in grey seals. Additional observational reports that described grey seal predation events in detail but were not accompanied by photographs/videos were classified as *possible* grey seal predation and were excluded from analysis.

### 2.2 Data analysis

All data processing and analyses were conducted in R (R Core Team, 2022), and maps were created in Manifold® System Release 8. The spatial distribution of grey seal predation strandings (all cases scored at least *possible*) in Scotland between 2010 and 2021 was mapped for grey seals, harbour seals and harbour porpoises. To account for spatial heterogeneity in data collection effort, non‐predation cases assigned to other causes of death, such as unlicenced shooting, starvation and pneumonia, were included. Changes to the proportion of the total number of strandings assigned to grey seal predation through time were assessed using a generalised linear model with a beta distribution. The model was run once for all prey species, including all strandings scored at least *possible* grey seal predation, and for seals a second time, more conservatively excluding *possible* cases and only retaining *definite*, *likely*, and *probable* grey seal predation. Data were tested for normality, and the statistical significance of changes through time was derived from *p*‐values.

To explore the spatial and temporal prevalence of grey seal predation at a finer scale, regional trends around Scotland (by Seal Monitoring Unit, SMU), calendar month and age class of seal carcasses were explored using odds ratios (Szumilas, 2010). These represented the odds that the cause of death was *definite*, *likely* or *probable* grey seal predation, against the odds that it was not (excluding *possible* cases). Odds ratios with 95% confidence intervals were calculated for each group (SMU, month, age class).

To investigate the level of specialisation within populations, individual predators were identified from grey seal predation observations through manual photographic identification (i.e. photo ID) following a protocol adapted from Langley et al. (2022), and capture histories of predation events were generated for each individual. For a subset of data from the Firth of Forth in the East Scotland SMU in 2019, strandings reports and observational accounts were combined in a capture–mark–recapture methodology to estimate the minimum number of predation events that were missed in the strandings data. Dead marine mammals may be missed in the strandings data if carcasses do not wash up, or if they are not discovered and reported to the strandings scheme. Indeed, the vast majority of seals that die at sea do not wash up, and the probability of strandings is also influenced by the cause of death (see Section 4 for further consideration into the caveats of strandings data). Grey seal predation events were considered potentially duplicated across the two datasets if there was a stranding that matched the description (e.g. prey species, age class) reported within seven days of an observation. Small marine mammals such as seals with a post‐mortem interval greater than 7 days usually have advanced decomposition, and so assigning cause of death is not possible (Ramos et al., 2024). Indeed, strandings with traumatic injuries that could be indicative of grey seal predation but with advanced decomposition are assigned *possible*, and *possible* cases were excluded from this analysis.

### 2.3 Leslie matrix

To examine the potential magnitude of the observed effect of grey seal predation on harbour seal populations, the results from the odds ratio analysis were used to build a female‐only age‐structured deterministic Leslie matrix for a stable population (Caswell, 2001; Leslie, 1945), and the effect of different predation scenarios was explored. Specifically, we modelled the population growth rate using a plausible range of vital rates (Arso Civil et al., 2019), assuming an equal sex ratio (Boulva & McLaren, 1979) and five age stages: pup (age 0), juvenile (age 1, 2 and 3) and adult (4+), which was an absorbing class. For the baseline model, we retained sets of vital rates that gave rise to a stable population (i.e. a growth rate of ±0.025; Hall et al., 2024).

Annual harbour seal abundance associated with the Firth of Tay and Eden Estuary SAC (East Scotland SMU) between 2010 and 2021 was estimated from scaling up August counts (Russell et al., 2022) using the proportion of the population estimated to be hauled out during the survey window, and thus available to be counted (Lonergan et al., 2013). Realistic predation scenarios were generated through an analysis of the sex ratio within the strandings data and scaling up the prevalence of grey seal predation in the strandings data to in part account for events likely missed (using the comparison with observational data). The change in population growth rate for each set of vital rates was calculated, and a hypothetical population of 100 individuals divided across a stable age structure was projected forward 10 years.

## 3 RESULTS

### 3.1 Analysis of strandings data

**FIGURE 1:**
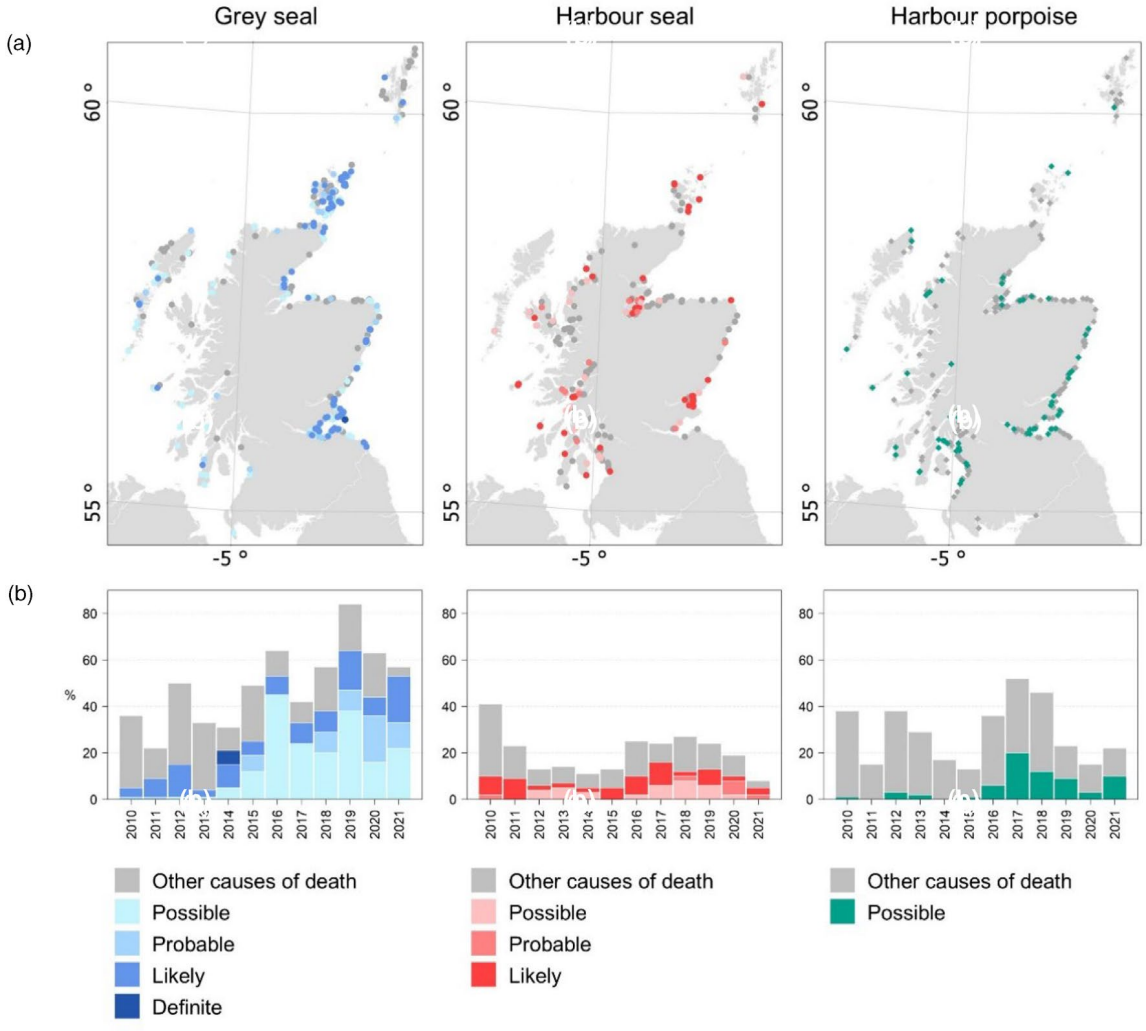
(a) Spatial distribution and (b) temporal trends of strandings cases in Scotland between 2010 and 2021 for grey seals (blue), harbour seals (red) and harbour porpoises (green) shaded by grey seal predation assessment score.

There was a total of 4226 seals reported to the Scottish stranding scheme between 2010 and 2021: 2434 grey seals, 787 harbour seals, 997 seals of indeterminate species and eight individuals from non‐resident species. Over the same period, there were 361 harbour porpoises reported. Cause of death was assigned to 588 grey seals and 242 harbour seals; the rest were mainly too decomposed or scavenged before being reported. In grey seals, 62% of assigned cases were at least *possible* grey seal predation (*definite*, *likely*, *probable* or *possible*): of these, 2% were classified as *definite* (*n* = 6), 32% *likely* (*n* = 116), 16% *probable* (*n* = 58) and 51% *possible* (*n* = 184). In harbour seals, 45% of assigned cases were at least *possible* grey seal predation: of these, none were classified as *definite*, 56% *likely* (*n* = 60), 11% *probable* (*n* = 12) and 33% *possible* (*n* = 36). There was also one hooded seal (*Cystophora cristata*) assigned *possible* grey seal predation. Of the 361 porpoise strandings, 19% (*n* = 67) were given the cause of death *possible* grey seal predation. The grey seal, harbour seal and harbour porpoise strandings classified as at least *possible* grey seal predation spanned the coastline of Scotland (Figure 1a).

The proportion of stranding cases assigned at least *possible* grey seal predation as a cause of death significantly increased over time for both grey seals and harbour seals (*p* < 0.001; Figure 1b). This trend was also significant when excluding *possible* cases for harbour seals (*p* < 0.05) but was not significant when using this more conservative grouping for grey seals (*p* > 0.05). While grey seal predation made up a smaller proportion of the causes of death assigned to harbour porpoise strandings in Scotland, the proportion of strandings assigned *possible* grey seal predation significantly increased between 2010 and 2021 (*p* < 0.001; Figure 1b).

**FIGURE 2:**
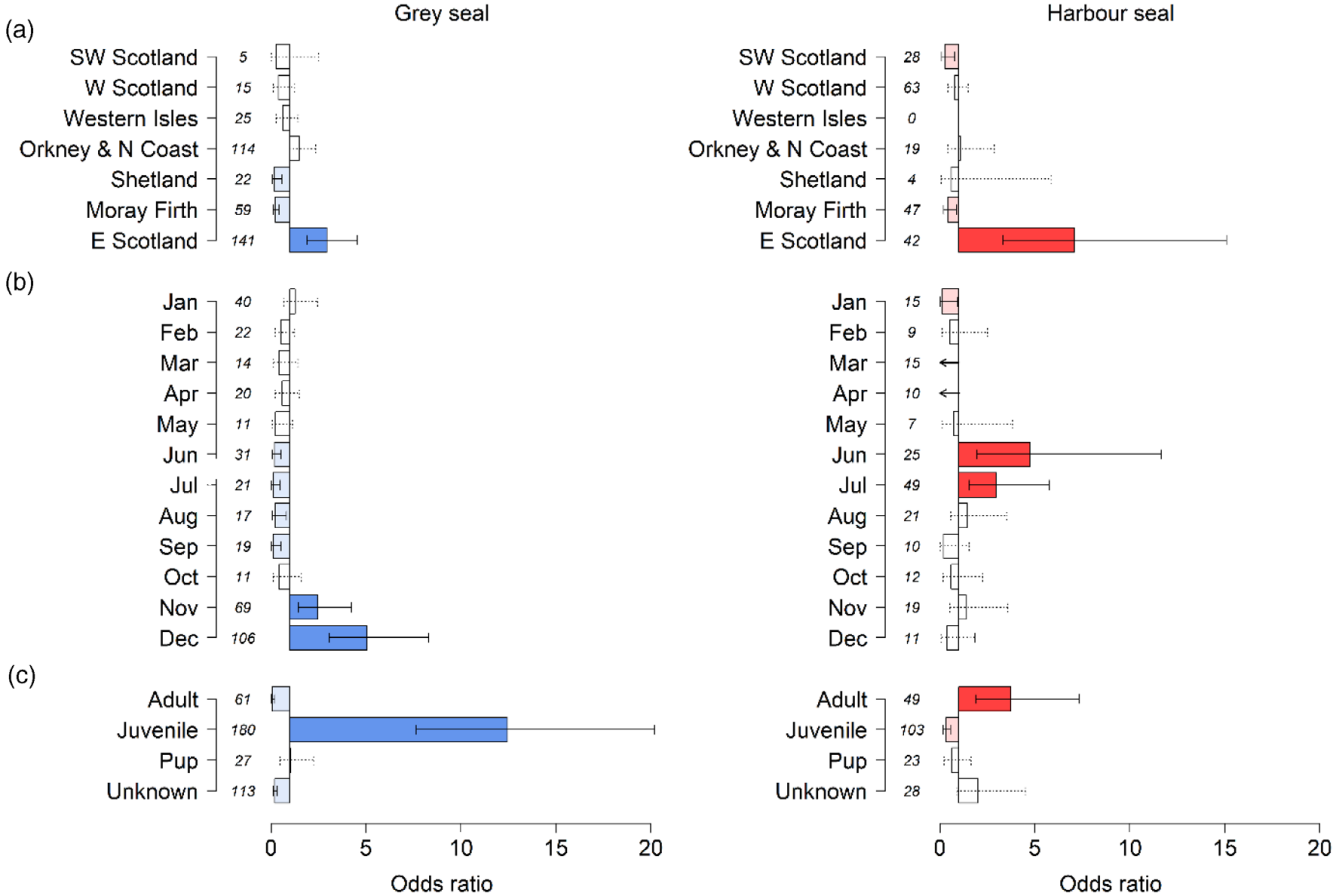
Odds ratios between *definite*, *likely* or *probable* grey seal predation against other causes of death for (a) Seal Monitoring Unit, (b) calendar month and (c) age class. Error bars represent 95% confidence intervals, dashed intervals spanning the null (OR = 1) show direction of odds was not significant and total sample sizes of all causes of death combined are presented in square brackets. Grey seals on the left‐hand side (blue) and harbour seals on the right‐hand side (red). Solid coloured bars represent positive odds (significantly greater than one), and shaded bars represent negative odds (significantly less than one).

Seal strandings had the greatest odds of being assigned *definite*, *likely* or *probable* grey seal predation as the cause of death in the East Scotland SMU (Figure 2a). The odds ratios for grey seal predation by calendar month differed between the two species (Figure 2b). Grey seals had the highest odds in the winter months of November and December, and harbour seals had the highest odds in the summer months of June and July. Age class also displayed an opposite pattern between grey and harbour seals (Figure 2c). Juvenile grey seals, including everything from weaned pups to subadults, and adult harbour seals had the highest odds of grey seal predation. Of the 38 adult harbour seals assigned *likely* or *probable* grey seal predation, 53% were female (*n* = 20), 29% male (*n* = 11) and 18% could not be sexed (*n* = 7); and at least 40% (*n* = 8) of the 20 adult females were pregnant.

### 3.2 Analysis of direct observations

Since 2014 there have been 30 observational reports of unique potential grey seal predation events in Scotland, from six of the seven SMUs: 21 in East Scotland (including those from Bishop et al., 2016), four in West Scotland, two in Orkney, one in Shetland, one in the Moray Firth and one in the Western Isles. Of these, 37% were classified as *definite* (*n* = 11) as the entire interaction including both the kill and feeding was captured, 47% were classified as *likely* (*n* = 14) and 18% were classified as *possible* (*n* = 5). From 25 *definite* and *likely* grey seal predation events, we identified seven adult male grey seals from good quality photographs. Five of the seven individuals were observed feeding on seals on more than one occasion, and the longest interval between two reported events involving the same individual was 6 years: August 2019 to March 2025. There was also one individual that was first observed preying on a young harbour seal in February 2022, and then 1 month later preying on a grey seal pup.

### 3.3 Complementing strandings data with direct observations

In 2019, there were six observational accounts of grey seal predation events reported from the Firth of Forth in the East Scotland SMU. Two of these cases involved the same individual male grey seal identified through photo ID. Prey species were also identified from these accounts, but a limited amount of prey pelage was visible, which precluded the individual identification of prey. Also in 2019, there were 17 stranding cases assigned *definite*, *likely* or *probable* grey seal predation from the same area. Two out of the six observations fell within the same 7 days as a grey seal predation stranding case. The other four observations were not potentially duplicated in the strandings data, indicating that the strandings data here underrepresented grey seal predation by at least 67%.

### 3.4 Leslie matrix

**FIGURE 3:**
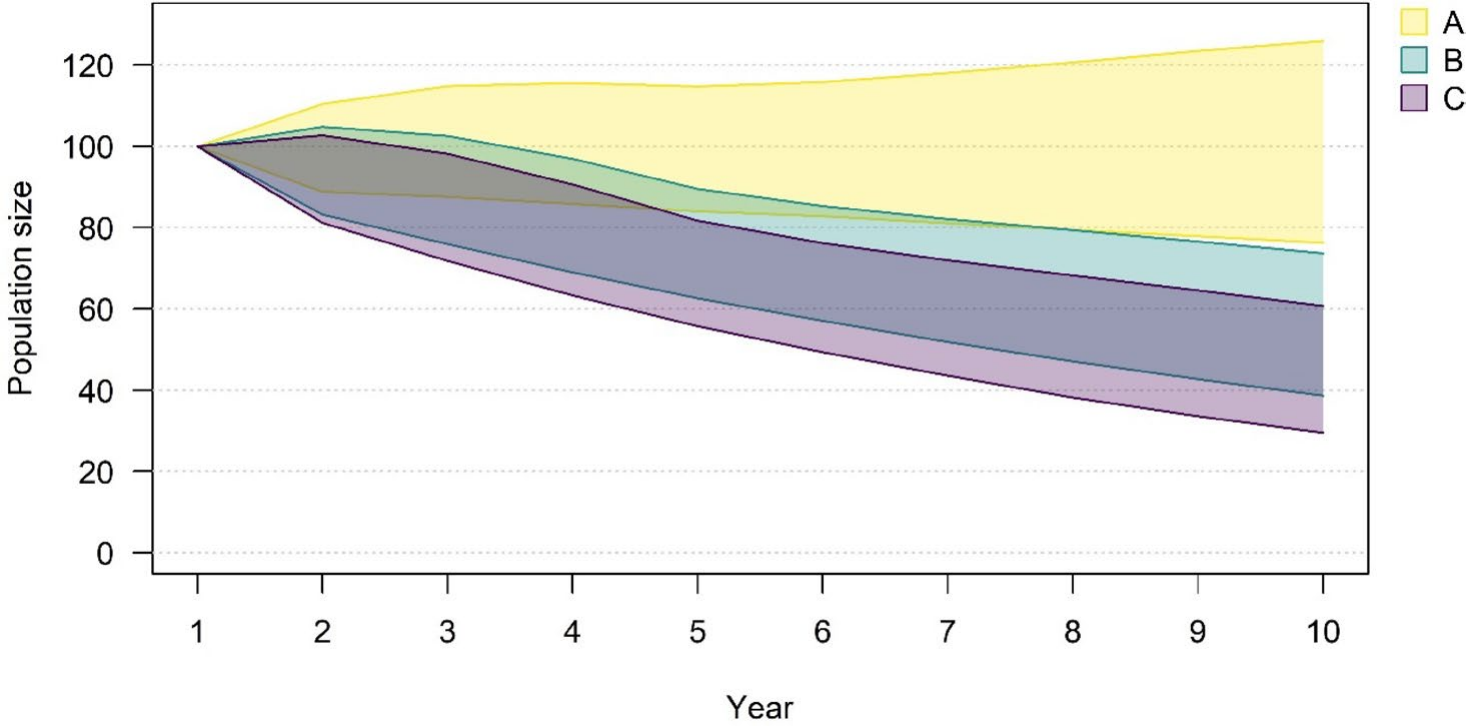
Population trends projected over 10 years using adjusted harbour seal growth rates for each predation scenario (A, B and C outlined in Table 2) showing 95% confidence intervals of all viable combinations of vital rates.

There was a slight female bias in the age‐specific predation strandings from the East Scotland SMU between 2010 and 2021, with the proportion of harbour seal cases that were female being 0.55 in adults, 0.56 in juveniles and 0.58 in pups. Based on the odds ratios, for every adult harbour seal in the strandings record, there were 0.1 juveniles and 0.16 pups. With this information, retained sets of vital rates which gave rise to a stable population were adjusted, and annual predation ratios for harbour seals in the Firth of Tay and Eden Estuary SAC were estimated (Table 1). Between 2010 and 2021, there were 34 harbour seal strandings assigned *likely* or *probable* grey seal predation in the SAC, 31 of which were adults. The predation ratio for adult females ranged from 0 to 0.11, which informed three predation scenarios (Table 2). The observed peak predation ratio for adult females in the strandings data, scaled up using the comparison between strandings and observations, resulted in a rate of change in the harbour seal growth rate of 7%–11%, well within the observed decline in the SAC (scenario C). Indeed, to generate the mean annual declines, the predation ratio would have to be 0.08 (scenario B; Figure 3). Acknowledging that the assumption of a constant and even sex ratio would likely be violated by female‐biased predation, we reran the model using the peak predation ratio across adult males and females (0.10) and confirmed that the rate of decline (7%–10%) would still be within the observed decline.

**TABLE 1:** Predation ratios calculated as the number of age and sex specific predation cases out of the estimated number of adult female harbour seals in the Firth of Tay and Eden Estuary SAC.

Year	Abundance			Predation strandings			Predation ratio
	August count^1^	Annual % decline	Adult females^2^	Adults^3^	Adult females^4^	Adjusted for minimum missed^5^	
2010	115	*NA*	80	8	4.4	7.3	0.09
2011	91	21	63	6	3.3	5.5	0.09
2012	73	20	51	6	3.3	5.5	0.11
2013	60	18	42	5	2.8	4.6	0.11
2014	51	15	35	1	0.6	0.9	0.03
2015	45	12	31	1	0.6	0.9	0.03
2016	42	8	29	2	1.1	1.8	0.06
2017	39	5	27	2	1.1	1.8	0.07
2018	38	3	26	0	0	0	0
2019	38	1	26	0	0	0	0
2020	38	0	26	0	0	0	0
2021	39	−1	27	0	0	0	0

*Note*: August counts^1^ are from aerial surveys (Russell et al., 2022) and the number of adult females^2^ was estimated assuming an equal sex ratio and adjusted for the estimated proportion of animals hauled out (divided by 0.72). Adult predation strandings^3^ are from the standings data, adjusted for a female bias (multiplied by 0.55)^4^ and missed cases (multiplied by 1.67)^5^.

**TABLE 2:** Rate of decline (expressed as 95% confidence intervals) in harbour seal growth rate based on three predation scenarios.

Scenario	Description	Predation ratio	Rate of decline (%)
A	No additional mortality caused by predation (baseline)	0	*NA*
B	Required predation to generate mean annual rate of decline in SAC 2010–2012 (8%)	0.08	5, 8
C	Observed peak predation in adjusted strandings data from the SAC	0.11	7, 11

## 4 DISCUSSION

While ubiquitous in nature, IGP is a rarely observed interaction between sympatric species that coexist within trophic levels. However, relatively few specialist individuals may have a significant effect on declining or already depleted populations. Here, we collated two unique datasets of stranded marine mammals and observational accounts to investigate adverse species interactions over a large spatial and temporal scale, highlighting the value of volunteer networks and citizen science. Our analysis provided insight into IGP as an apparent specialism exhibited by multiple individuals across distinct subpopulations. This study highlights the significance of rarely observed species interactions, such as IGP, in shaping population dynamics and provides evidence for its potential role in hindering the recovery of sympatric species populations.

When fatal species interactions such as IGP first emerge in populations, we tend to assume that they represent aberrant behaviour involving rogue individuals (Bédard et al., 1993; Rohner et al., 2020). In this study, which builds upon Bishop et al. (2016) and Brownlow et al. (2016), we demonstrated that IGP and cannibalism are exhibited repeatedly by individual grey seals found across populations, at levels that may cause population impacts on prey species. In one previously published case study, a single adult male was linked to 14 grey seal pup carcasses over a 10‐day period (Bishop et al., 2016; Brownlow et al., 2016). Here, we were able to link one individual to both grey seal and harbour seal prey, and another individual to predation events almost 6 years apart. This is the first evidence that grey seal cannibalism and IGP of harbour seals are directly linked, and that it is a repeatable behavioural trait exhibited by a limited, but potentially increasing, number of individuals. Furthermore, our photo ID catalogue of known marine mammal predating grey seals is only a minimum estimate for the prevalence of this strategy across grey seal populations. Generally, repeatability in measurable phenotypes is a population‐level measure, but at the individual level represents intraspecific variation in phenotypes, or individual specialisation (Devictor et al., 2010). Interestingly, individual specialisation is considered an adaptive strategy to reduce pressures imposed through competition and effectively respond to fluctuations in resource availability (Araújo et al., 2011).

Unlike traditional predator–prey interactions, including other marine mammal predating specialists such as killer whale ecotypes (Ford et al., 1998), the recovered carcasses in grey seal predation cases indicate that very little blubber is ingested (Brownlow et al., 2016). Indeed, individual predators have been observed only removing a small section of blubber before abandoning the carcass and catching further prey (Bishop et al., 2016). This may be related to the force required to tear the skin/blubber layer when the carcass is cold (D. Thompson, *personal communication*). Furthermore, feeding exclusively on seal blubber would be unlikely to provide sufficient nutritional benefit to predators (Brunborg et al., 2006), and mammals with high‐fat diets can develop hyperlipidaemia (Bertin, 2023). This indicates that marine mammal predation by grey seals is only supplementing diet.

IGP and cannibalism had the highest odds of cause of death in grey and harbour seals during their respective breeding seasons. Breeding in both species represents a time of year when animals aggregate in and around breeding colonies, making individuals somewhat spatially constrained (Fedak & Anderson, 1982; Thompson et al., 1994). The grey seal breeding season in winter also represents a time of year when the density and abundance of young animals are highest. Whereas in the summer, aggregations of breeding and moulting harbour seals would provide optimal foraging opportunities to specialist predators. This is also the case in the Pacific, where harbour seals are more vulnerable to white shark (*Carcharodon carcharias*) predation during their annual moult in July (Anderson et al., 2008). Grey seal predation of harbour seals in Scotland was also reported to be low in spring, which encompasses the grey seal moult, when grey seals spend long periods of time hauled out to reduce heat loss during this thermally energetic period (Schop et al., 2017).

Often, predators target a subset of prey populations which can have different effects on population survival and fecundity (Cherry et al., 2016; Fu et al., 2001). Indeed, cannibalism is most common in early life‐stages (Ivanov et al., 2020; Ryazanov et al., 2017), and size dimorphism is also common in examples of IGP (Fedriani et al., 2000; Petty et al., 2003). Here, it was evident that juvenile grey seals were most vulnerable to cannibalism. This is likely biased towards weaned pups, who remain on breeding colonies unattended and relatively immobile for up to 4 weeks as they undergo a post‐weaning fast (Reilly, 1991). Furthermore, pups of the year are more likely to be inshore as they develop effective foraging strategies (Carter et al., 2017), and so strandings are possibly biased towards younger seals. Pupping results in a large temporary influx of grey seal juveniles; over 50% of which will die in their first year (Thomas et al., 2019). Indeed, in many regions of Scotland, the grey seal breeding populations are near carrying capacity, with density dependence likely acting through prey‐limited pup survival, reducing first‐year survival to <15%. As such, removing such seals through predation is unlikely to have a population‐level effect.

Analysis of the strandings data in Scotland demonstrated that it was the adult age class in harbour seals that had the highest odds of predation against other causes of death. This may be influenced by the breeding strategy of harbour seals, where females give birth in or close to the water and have restricted range in early lactation (Bowen et al., 1999; Thompson et al., 1994). Harbour seals are not markedly sexually dimorphic, and both sexes are smaller than adult male grey seals. Strandings were slightly female‐biased, which is significant for harbour seal populations as sexually mature females have high reproductive value (Fisher, 1930). Furthermore, at least 40% of predated female harbour seals were pregnant, and non‐pregnant females could have recently given birth to pups that would fail to successfully wean. This means that a single grey seal predation event can directly remove more than a single individual, before even considering the reduction in future reproductive output of the population.

The effect of IGP on harbour seal populations was explored in a Leslie matrix. We focused on a small population of harbour seals in the Firth of Tay and Eden Estuary SAC which had already declined by ~80% over the 10 years prior to this study (Russell et al., 2022). In this depleted population, the effect of predation was likely greater than in a larger population (i.e. the rate of predation is not linear, and one specialist predator can remove a higher proportion of a smaller population). Nevertheless, the magnitude of the decline was replicated by the level of predation estimated in the SAC. The simulated predation scenarios were informed by strandings data and observational accounts, but the reduction in survival could have been caused by multiple, interacting drivers. These include exposure to biotoxins such as domoic acid (Hall et al., 2024), and/or prey competition with grey seals (Langley, 2024). However, the level of IGP recorded within the SAC would cause further declines.

It is also possible that harbour seals in some regions of Scotland could be in a ‘predator pit’, where grey seal predation is preventing recovery of already depleted populations (Grange et al., 2004; Swain & Benoît, 2015). Indeed, deterministic models investigating the predator–prey interactions between wolves (*Canis lupus*) and elk (*Cervus canadensis*) demonstrated that predator pits emerged when predation was highly stochastic, and predator populations had high carrying capacities (Clark et al., 2021). The relationship between grey seals and harbour seals is further complicated by the influence of interspecific competition, and so it is possible that increasing grey seal densities could be holding harbour seals in a ‘competitor pit’, preventing recovery of populations that fall below a certain density threshold (Cabezas‐Díaz et al., 2011). Here, it is difficult to separate out the effects of grey seals on harbour seal populations, but other study systems have found that it is the interactions between predation and food availability that drive population cycles (Krebs, 1996).

Seal population densities may also partly explain why reported incidences of IGP and cannibalism were highest in the East Scotland SMU. During the winter breeding season, large numbers of grey seals aggregate at breeding colonies in the region: Fast Castle (~4500 pups) and the Isle of May (~2000 pups; Russell et al., 2022). Greater densities of grey seals increase the probability of direct interactions, both with conspecifics and with interspecific competitors. Indeed, high densities at pinniped breeding colonies have been associated with higher pup mortalities (Coulson & Hickling, 1964; Harcourt, 1992), and in terrestrial carnivore populations, dense aggregations in foraging areas have been shown to increase agonistic interactions, including cannibalism (Newsome et al., 2019). In this study, we were able to account for some spatial bias in the reporting of strandings by incorporating non‐predation strandings cases as a baseline for effort and assessing the prevalence of predation compared to other causes of death.

The proportion of grey seal predation cases in the Scottish strandings record increased through time between 2010 and 2021. This significant increase was more marked for grey seal cannibalism rather than IGP of harbour seals or harbour porpoises. Grey seal cannibalism compared to other causes of death in grey seals increased by almost three‐fold between 2015 and 2016, possibly associated with increased awareness after a seminal event in the winter of 2014 that received publicity (Bishop et al., 2016; Brownlow et al., 2016). Reports of direct observations also correlated with increased publicity of the project. This is an inherent bias in opportunistic surveillance and citizen‐science data but will be minimised with continued effort to engage members of the public through scientific outreach.

Although the numbers of grey seal predation cases in the standings record underrepresent the level of grey seal predation within the population, the magnitude of grey seal predation compared to other causes of death is overrepresented in strandings data. In Scotland, regional grey seal populations are approaching carrying capacity and it is predicted that >30,000 grey seal pups die each year (Thomas et al., 2019). However, a very small proportion of these pups wash up, and even fewer are discovered and reported within a short enough timeframe to confidently assign cause of death. Mortalities that occur on haulout sites and around densely aggregated breeding colonies, such as those caused by grey seal predation, are therefore more likely to be represented. Conversely, other causes of death such as starvation may be more likely to occur further offshore as animals struggle to search for food. Stranding probability is influenced by many factors such as oceanographic topography, wind direction, water temperature and also cause of death, which can affect the rate of decomposition and buoyancy (Peltier et al., 2012). This presents challenges in estimating the rate of missed mortalities for different causes of death within the strandings data, and results therefore should be interpreted with this caveat in consideration.

This study was a first attempt to quantify both the prevalence of grey seal IGP within populations and its effect on intraguild prey populations. The drivers behind the emergence of IGP and cannibalism remain unclear, but one hypothesis proposed to explain Southern sea lion (*Otaria flavescens*) predation of South American fur seals (*Arctocephalus australis*) is sexual frustration (Harcourt, 1993). Indeed, there is evidence from the Wadden Sea of coercive copulation between grey seals and pregnant female harbour seals resulting in abortion, haemorrhaging and death (Rohner et al., 2020). There were no pathological attributes linked to coercive copulation in this study, but carcasses did not routinely undergo full necropsies, so it cannot be ruled out. Other hypotheses include density‐related aggression (Ryazanov et al., 2017) and increased breeding success (Bassar et al., 2023). However, IGP must also provide some nutritional benefit, which is the reported motivation behind interspecific IGP in leopard seals (*Hydrurga leptonyx*; Boveng et al., 1998), Southern elephant seals (*Mirounga leonina*; Penry et al., 2013) and otariids (Baylis et al., 2023; Harcourt, 1993; Mathews & Adkison, 2010; Robinson et al., 1999; Ryazanov et al., 2017). To our knowledge, there have been no observations of female grey seals predating marine mammals. IGP in other pinniped species is exhibited almost exclusively by males, with the one exception being leopard seals where adult females are known to predate the pups of Antarctic fur seals (*Arctocephalus gazella*; Hiruki et al., 1999). Interestingly, leopard seals are also unique in their female‐biased sexual dimorphism (Kienle et al., 2022), which would suggest that size rather than sex may be an important driver. This study found a peak in cannibalism during the grey seal breeding season, which could be further investigated by a paternity analysis to explore whether feeding on pups enables adult males to stay at breeding colonies longer and sire more offspring (e.g. Worthington Wilmer et al., 1999). It also remains unclear as to whether this behaviour develops through social learning (Schakner et al., 2016). However, this study found ‘blubber‐eating’ grey seals present across populations, and the individual and opportunistic nature of grey seals lends support to the behaviour emerging independently. From our study, it is difficult to see how small and already depleted populations of harbour seals could recover alongside large and, in some places, increasing grey seal densities.

## AUTHOR CONTRIBUTIONS

Izzy Langley, Andrew Brownlow and Debbie J. F. Russell conceived the ideas and designed methodology; Izzy Langley, Andrew Brownlow and Debbie J. F. Russell collected the data; Izzy Langley and Andrew Brownlow analysed the data; Izzy Langley led the writing of the manuscript. All authors contributed critically to the drafts and gave final approval for publication.

## CONFLICT OF INTEREST STATEMENT

The authors have no conflicts of interest to declare.

## Data Availability

The data underpinning this research can be accessed at the University of St Andrews Data Repository (https://doi.org/10.17630/6a0d8b02‐1cca‐4d2a‐8949‐d658c7097cd3).
